# Clinical practice of prone position and nutritional support using magnetic navigation-guided nasoenteric tube placement in a patient with prone position contraindications: A case report

**DOI:** 10.1097/MD.0000000000045263

**Published:** 2025-10-31

**Authors:** Qianrong Ding, You Yuan, Jing Yang, Lican Zhao, Huan Liu, Yongming Tian

**Affiliations:** a Department of Critical Care Medicine, West China Hospital, Sichuan University/West China School of Nursing, Sichuan University, Chengdu, China; b Department of Critical Care Medicine, Affiliated Hospital of Zunyi Medical University, Zunyi, Guizhou, China.

**Keywords:** ARDS, contraindications, magnetic navigation, nasoenteric tube, pelvic fractures, prone positioning ventilation

## Abstract

**Rationale:**

Prone position ventilation (PPV) has been shown to improve oxygenation and reduce mortality in patients with acute respiratory distress syndrome; however, its use in patients with contraindications to the prone position is challenging. Additionally, patients who undergo PPV after laparotomy are often associated with gastrointestinal dysfunction. Therefore, early enteral nutrition is crucial, but effective bedside tube placement techniques are currently lacking in clinical practice.

**Patient concerns:**

A 57-year-old male patient developed acute respiratory distress syndrome and pelvic fractures without internal fixation after multiple injuries. He had contraindications to PPV and experienced gastrointestinal dysfunction and enteral nutrition issues following laparotomy. Traditional methods cannot address both problems at the same time.

**Diagnoses:**

The primary diagnoses include multiple trauma, complex intra-abdominal infection, pulmonary contusion, multiple rib fractures, pelvic fractures, and septic shock.

**Interventions:**

The medical team used a burn rotation bed for PPV and employed magnetic navigation technology to guide the placement of a double-lumen nasoenteric tube for enteral nutrition.

**Outcomes:**

The patient’s oxygenation improved, with smooth sputum drainage and reduced pulmonary inflammation. Gastrointestinal function was restored, and enteral nutrition was successfully initiated. Subsequently, the patient underwent successful pelvic fixation surgery and was transferred out of the intensive care unit. The patient was eventually discharged and continued to be followed up as an outpatient.

**Lessons:**

This case demonstrates the feasibility of implementing PPV and nutritional support despite contraindications through careful clinical decision-making, which involves weighing the risks and benefits, and offers valuable clinical experience for similar cases.

## 1. Introduction

Patients with multiple injuries often develop acute respiratory distress syndrome (ARDS) due to systemic inflammatory response syndrome and multiple organ dysfunction syndrome caused by severe trauma.^[1–3]^ As a common clinical type of acute respiratory failure, the pathophysiological features of ARDS are mainly characterized by disruption of the alveolar-capillary barrier, decreased lung compliance, and intractable hypoxemia.^[4]^ In recent years, a large body of evidence-based medical evidence has shown that prone position ventilation (PPV) can significantly improve the oxygenation index and reduce the 28-day mortality rate in patients with moderate-to-severe ARDS.^[5,6]^ This is achieved by improving the ventilation-to-blood flow ratio, attenuating alveolar inhomogeneity, and decreasing ventilator-associated lung injury.^[7]^

However, for patients with contraindications to PPV, such as those with an unstable pelvis, recent abdominal trauma, or those who have undergone laparotomy, the implementation of this treatment poses many challenges.^[8]^ In addition, critically ill patients undergoing PPV often experience gastrointestinal dysfunction due to analgesic sedation and positional changes. Given the increased risk of aspiration with PPV, the ESPEN guidelines recommend postpyloric feeding, primarily through jejunal feeding.^[9,10]^ The high metabolic state following trauma necessitates the early implementation of enteral nutrition.^[11,12]^ However, the positional adjustment (right lateral recumbency) required for traditional nasoenteric tube placement may cause secondary injury, thereby increasing the difficulty and risk in clinical practice.

This case report, through a specific case, explores how to successfully achieve PPV when prone positioning is contraindicated: by safely implementing prone ventilation using a burn rotation bed and by precisely placing a nasoenteric tube without changing the patient’s position.

## 2. Patient information

The patient, a 57-year-old man weighing 65 kilograms and standing 162 centimeters tall, has a body mass index of 24.8 and works as a laborer. While lifting a glass, he injured his chest and abdomen when the lifting rope snapped. He felt immediate abdominal pain but showed no signs of syncope, nausea, or vomiting. According to his family, he had a splenectomy 13 years ago. His medical history was otherwise unremarkable, with no chronic or underlying conditions, no hereditary disorders, and stable psychosocial status. After the injury, he was promptly admitted to a local hospital. He underwent emergency procedures including small bowel resection, repair of mesenteric laceration, repair of colonic serosal tear, adhesiolysis, and debridement of the left hip open wound, along with placement of nasogastric, abdominal, and thoracic drainage tubes. After 5 days without clinical improvement, his family transferred him to West China Hospital.

**Figure 1.**
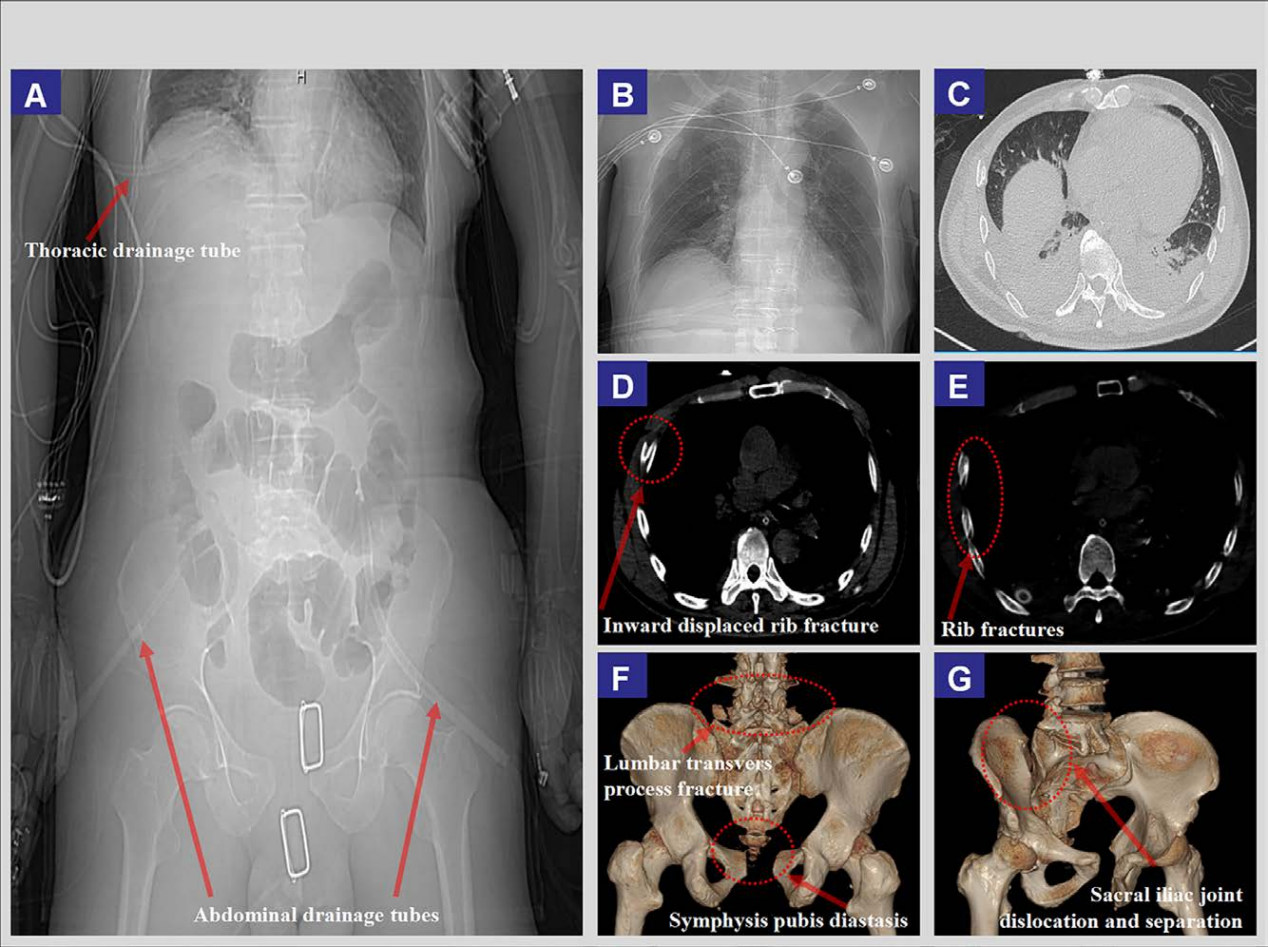
Emergency CT imaging data of the patient with multiple trauma. CT = computed tomography.

The emergency computed tomography scan revealed (Fig. 1A–G): mild distension of the intestinal loops in the abdomen; bilateral transverse process fractures of the L5 vertebra, anterior fracture of the S3 vertebra, and pubic symphysis diastasis; multiple rib fractures, with significant displacement of the right 4th to 6th ribs; right chest tube in situ, with small pleural effusion on the left side; partial atelectasis and consolidation in the lower lobes of both lungs, a bulla in the right upper lobe, small pericardial effusion, and no abnormalities in the brain parenchyma. The patient was subsequently transferred to the intensive care unit (ICU) for treatment. The primary diagnoses were polytrauma; complex intra-abdominal infection; pulmonary contusion; multiple rib fractures; pelvic fractures; sepsis; and hypoalbuminemia.

Upon admission to the ICU, the patient was placed on mechanical ventilation (FiO₂: 70%, VT: 480 ml, PEEP: 8 cmH₂O). Analgesic and sedative therapy was administered via intravenous infusion of remifentanil at 2 to 4 μg/kg/h and midazolam at 0.2 to 0.4 mg/kg/h. In contrast, norepinephrine at 24 μg/kg/h was used to maintain circulation. The Richmond Agitation-Sedation Scale score was −1 to −2, the Critical-Care Pain Observation Tool score was 0, the Braden Scale for Predicting Pressure Sore Risk was 11, the Morse Fall Scale score was 65, and the Barthel Index was 0. The Nutritional Risk Screening 2002 score was 5, indicating the presence of nutritional risk. Given that the patient had undergone intestinal resection and gastrointestinal function had not yet recovered, the medical team decided to withhold enteral nutrition temporarily and only perform gastric decompression, which resulted in the drainage of 400 to 600 mL of gastric fluid daily.

## 3. Timeline

**Figure 2.**
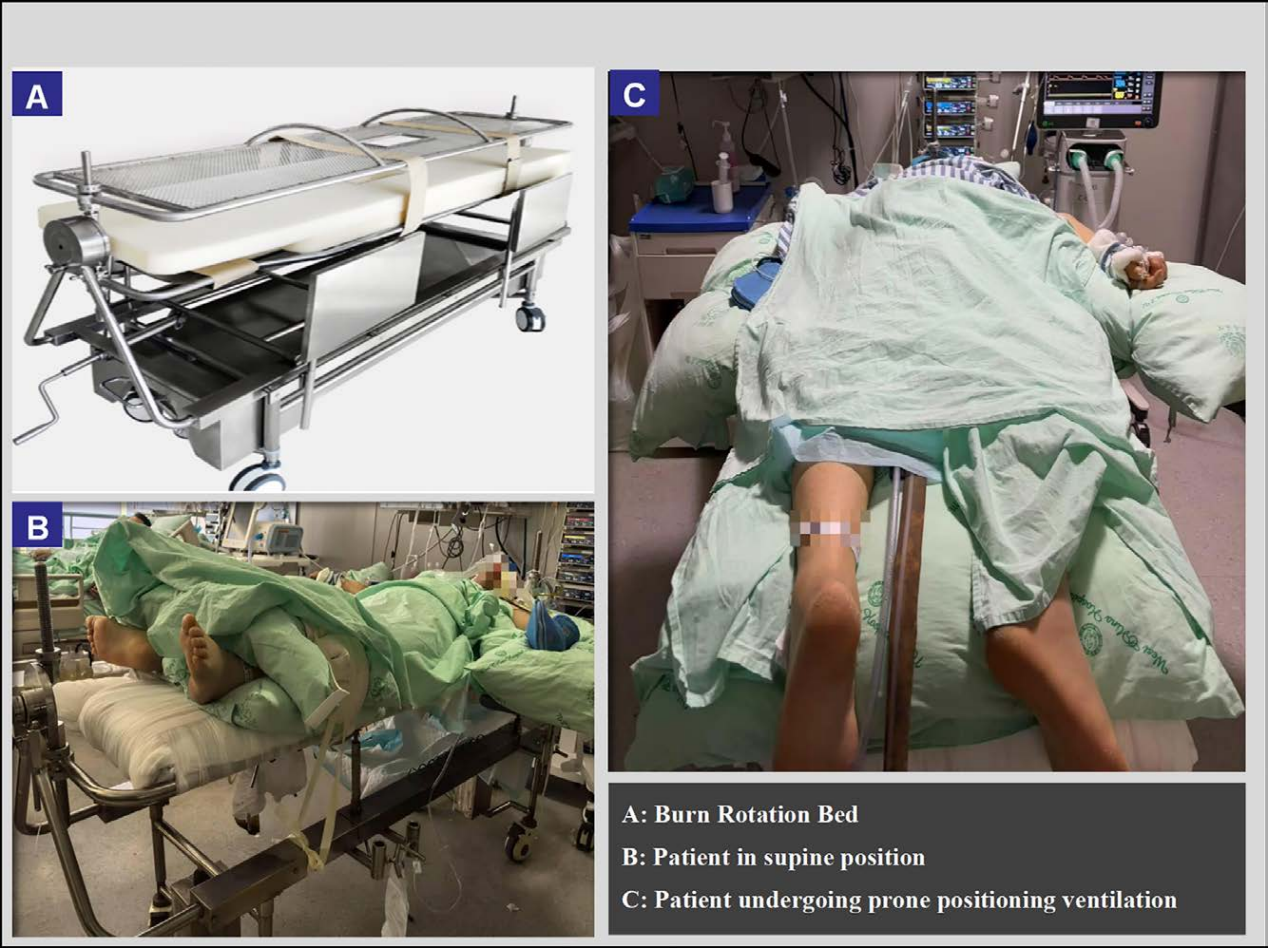
Implementation of prone positioning ventilation with the aid of a burn rotation bed.

On ICU day 2, the patient developed moderate ARDS (PaO₂/FiO₂ 139 mm Hg). Despite contraindications due to unstable pelvic fractures without internal fixation, the multidisciplinary team opted for prone positioning using a specialized burn rotation bed (Fig. 2A–C) after a risk-benefit analysis. This intervention achieved dual benefits: enhanced pulmonary secretion clearance and consequent inflammatory improvement, as well as significant oxygenation enhancement, with a 89.1% increase in PaO₂/FiO₂ (from 139 to 262.8 mm Hg) over 72 hours.

**Figure 3.**
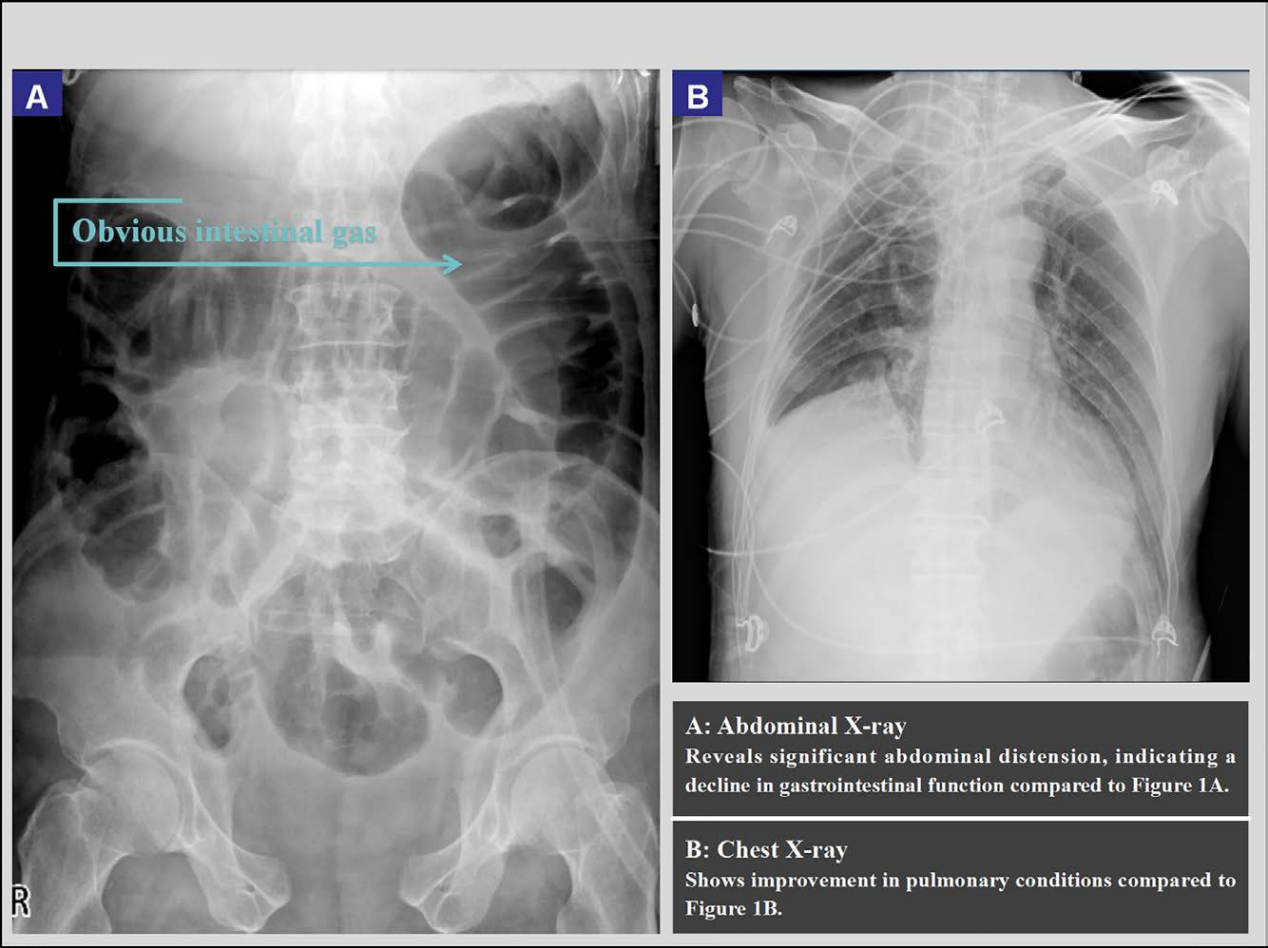
Day 2 prone ventilation abdominal and thoracic imaging.

**Figure 4.**
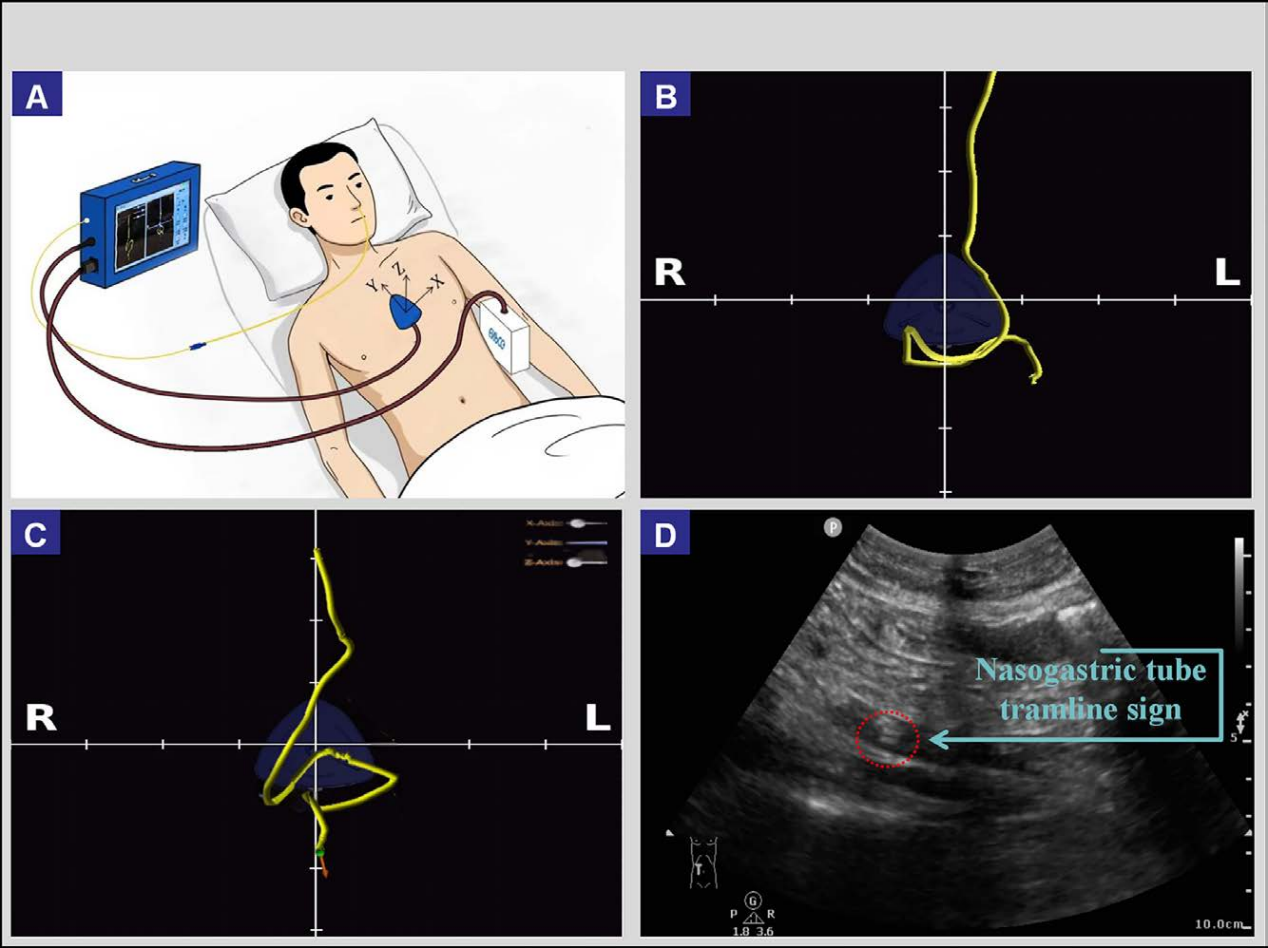
Principle and positioning of magnetic navigation-guided nasogastric tube insertion.

On ICU day 4, bedside X-ray imaging (Fig. 3A–B) indicated that PPV had achieved some effect, but the pulmonary condition had not fully recovered, and gastrointestinal bloating had worsened. The patient continued to face the dual challenges of gastrointestinal bloating and nutritional deficiency. The medical team decided to use 3-dimensional directional electromagnetic navigation technology to guide the placement of a double-lumen nasoenteric tube (one lumen in the stomach for gastrointestinal decompression and the other lumen at the jejunal level for drug administration and enteral nutrition support). This technology creates a 3-dimensional coordinate system (X, Y, Z) using a magnetic field generator and a locator. The path of the nasoenteric tube, guided by a sensor-equipped wire, is tracked in real-time and displayed as a 3-dimensional graphic (Fig. 4A). Due to abdominal bloating limiting ultrasound localization, the team adjusted the Y-axis based on spatial positioning to confirm the location (Fig. 4B-C). Video 1 in the attachment provides a detailed demonstration of the procedure for nasoenteric tube placement guided by electromagnetic navigation. After administering a laxative, the patient passed loose stools, and gastrointestinal function was restored. The next day, an ultrasound reexamination was performed to reconfirm the position (Fig. 4D). Subsequently, trophic enteral nutrition was initiated through the jejunal lumen, while gastric decompression continued through the gastric lumen to prevent excessive accumulation of gastric fluid.

## 4. Follow-up and outcomes

**Figure 5.**
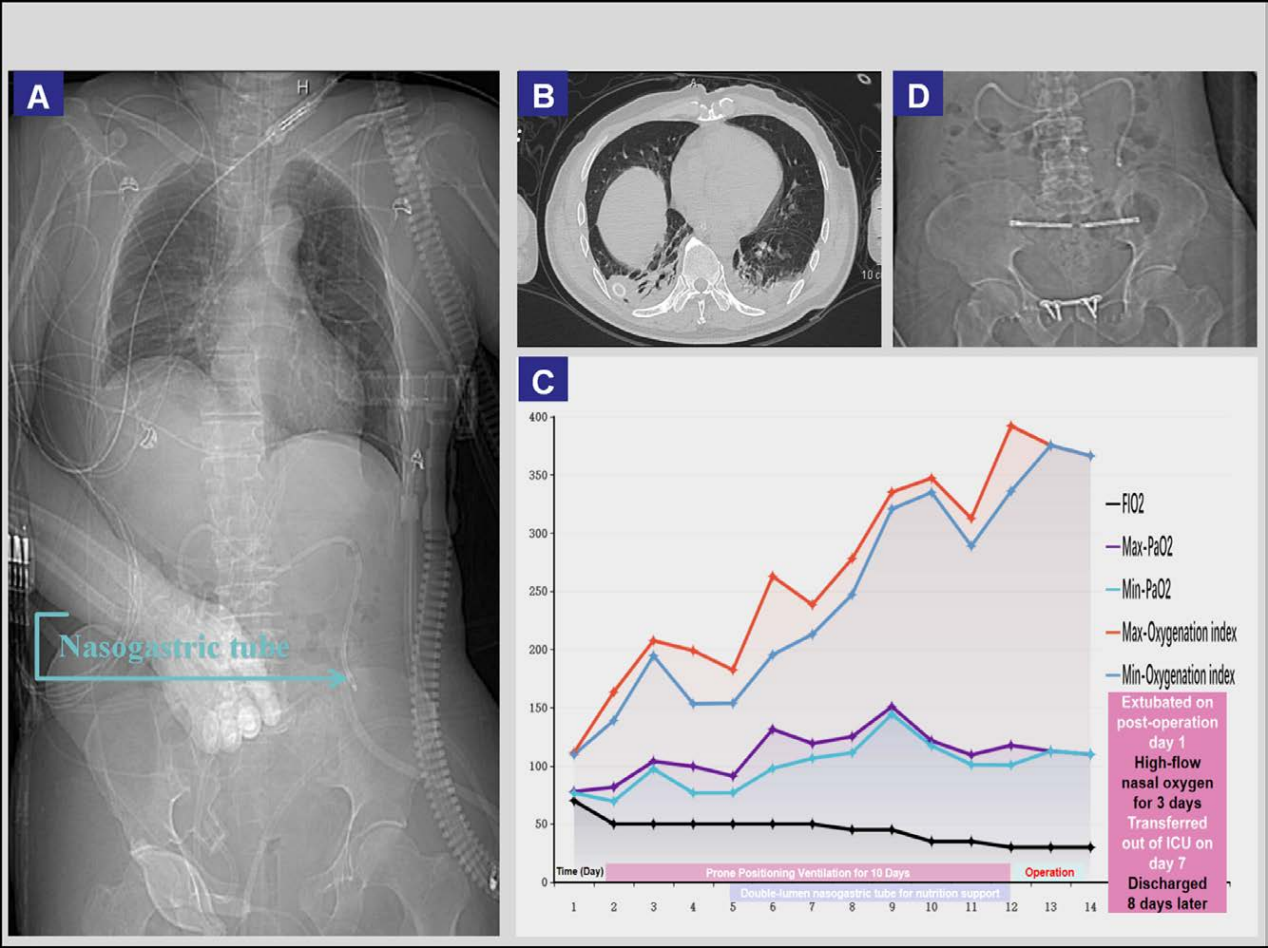
Imaging and oxygenation index outcomes after prone ventilation and enteral nutrition.

After 10 days of PPV, 7 days of enteral nutrition support, and other comprehensive treatments, imaging results revealed (Fig. 5A–B) that the patient had met the preoperative criteria for pelvic and sacroiliac joint fixation surgery. The PPV significantly improved the oxygenation index (Fig. 5C). The patient successfully underwent pelvic and sacroiliac joint fixation surgery (Fig. 5D). As shown in Figure 5A and 5D, the nasojejunal tube passed through the duodenal ligament to reach the jejunal segment, which demonstrates that electromagnetic navigation-guided placement of the nasojejunal tube is more advantageous than X-ray positioning. On the day following surgery, the patient was weaned off the ventilator and started on high-flow nasal cannula oxygen therapy. On the third day, the patient’s swallowing function recovered, and oral intake of liquids was initiated. On the 7th day, the patient’s condition stabilized, and he was transferred out of the ICU to resume a regular diet. The patient was discharged 8 days later after making a full recovery.

## 5. Discussion

This case report presents the management strategy for a patient with multiple trauma, pelvic fractures, and ARDS. The core challenges included how to implement PPV in the presence of contraindications due to pelvic instability and how to address gastrointestinal dysfunction and safely and effectively deliver enteral nutrition during prone positioning. The medical team successfully overcame these challenges using a burn rotation bed and electromagnetic navigation-guided placement of a double-lumen nasoenteric tube, providing valuable insights for similar cases.

### 5.1. The necessity and challenges of implementing PPV in ARDS patients with contraindications

In 2024, the global definition of ARDS was updated in the American Journal of Respiratory and Critical Care Medicine. In addition to updating the oxygenation criteria, incorporating new respiratory support modalities, adding ultrasound diagnosis, and enhancing applicability in resource-limited settings, the new definition emphasizes that ARDS is caused by acute, diffuse, inflammatory lung injury. This injury leads to increased alveolar-capillary permeability and reduced ventilated lung tissue, resulting in hypoxemia and bilateral pulmonary edema. Based on the oxygenation index, ARDS is classified into mild (200 mm Hg < PaO₂/FiO₂ ≤ 300 mm Hg), moderate (100 mm Hg < PaO₂/FiO₂ ≤ 200 mm Hg), and severe (PaO₂/FiO₂ ≤ 100 mm Hg) categories.^[4,13,14]^ For patients with moderate-to-severe ARDS and a PaO₂/FiO₂ ratio < 150 mm Hg and PEEP > 5 cmH₂O, if optimizing ventilator settings and low-tidal-volume lung-protective ventilation fail to improve oxygenation, PPV is recommended by both the 2024 American Thoracic Society and 2023 European Society of Intensive Care Medicine guidelines for managing ARDS.^[8,15,16]^

PPV has been widely demonstrated to improve oxygenation and reduce mortality in patients with ARDS significantly. It optimizes lung volume distribution, reduces alveolar collapse, improves the ventilation-perfusion ratio, alleviates the inflammatory response, and promotes drainage of secretions, thereby considerably enhancing oxygenation and respiratory function in ARDS patients and positively impacting the circulatory system. The longer the duration of PPV, the better the potential outcomes may be. However, absolute contraindications for PPV include unstable spinal fractures and late pregnancy. In contrast, relative contraindications include hemodynamic instability, unstable pelvic or long bone fractures, open abdominal wounds, and elevated intracranial pressure.

The patient had pelvic fractures (pubic symphysis separation and sacroiliac joint dislocation) that contraindicated prone positioning and had not undergone internal fixation, posing significant challenges for PPV. Despite these contraindications, the patient had bilateral pulmonary infiltrates and a persistent oxygenation index of <150 mm Hg. After a comprehensive assessment, the medical team determined that the potential benefits of PPV outweighed the risks. To safely and effectively implement PPV, the team first used a fixation belt to immobilize the fracture site. It employed a burn rotation bed to achieve “whole-body turning,” avoiding localized pressure on the pelvis. They continuously monitored pelvic stability and provided appropriate analgesia and sedation as needed. During the transition to the prone position, the team closely monitored for potential complications. Over the 10 days of PPV, the patient did not experience vomiting, choking, accidental tube dislodgement, tracheal tube displacement or obstruction, hemodynamic instability, brachial plexus injury, or pressure ulcers, and achieved a favorable outcome.

### 5.2. The necessity and challenges of inserting double-lumen nasojejunal tubes in ARDS patients at high aspiration risk during PPV

ARDS patients on prolonged mechanical ventilation face high risks of aspiration, increased catabolism, and significant energy expenditure, making enteral nutrition support essential. As recommended by both the ESPEN and ASPEN guidelines for critically ill patients, early enteral nutrition is strongly encouraged to maintain nutritional balance, prevent muscle wasting, and improve clinical outcomes.^[10,17]^ However, PPV and the use of analgesics and sedatives can increase gastrointestinal compression, leading to higher intra-abdominal pressure and delayed gastric emptying, which in turn raises the risks of feeding intolerance and may cause aspiration.^[12,18]^ Additionally, patient repositioning may cause displacement or dislodgement of the nasoenteric tube. As shown in Figure 1A and Figure 3B, the longer the duration of prone ventilation and the higher the doses of sedatives, muscle relaxants, and vasopressors, the more pronounced the impact on gastrointestinal function. Given that the patient had undergone intestinal surgery 11 days prior, early dual-lumen nasoenteric tube trophic feeding combined with continuous gastric negative pressure drainage was implemented to promote gastrointestinal motility and alleviate bloating.

In clinical jejunal nutrition practice, there is a lack of efficient bedside techniques for placing nasoenteric tubes. Traditional blind insertion methods, which rely on operator experience and patient physiological responses, often result in inaccurate tube placement. This necessitates X-ray confirmation, which increases the risks associated with patient movement (such as fracture), medical costs, feeding delays, and radiation exposure.^[19]^ While endoscopic and gastrointestinal contrast imaging techniques improve accuracy, they are technically demanding, costly, and require patient transfer, further elevating safety risks.^[20]^ Ultrasound-guided techniques reduce reliance on X-rays but demand high operator skill, typically require 2 professionals, and are susceptible to interference from factors such as pneumoperitoneum, intestinal gas, and obesity.^[21]^

To overcome these challenges, the medical team implemented bedside magnetic navigation-guided placement of nasoenteric tubes. This technique establishes a 3D coordinate system using a magnetic field generator and locator to track the tube path in real-time (Fig. 4B and C for 3D path visualization; a detailed video is attached). The method successfully achieved prone-position ventilation and nutritional support. Compared to traditional methods, magnetic navigation is more straightforward to operate, more precise, and offers greater timeliness. It could better replace X-ray positioning, potentially becoming a new standard for tube placement.^[22]^

## 6. Innovation and clinical significance

This case innovatively implemented PPV, typically contraindicated, marking a first in medical literature, especially with unfixed pelvic fractures. It demonstrates that prone ventilation, combined with nutritional support, is not only feasible but also practical, following a careful risk-benefit analysis. Using a custom burn rotation bed, the medical team safely positioned the patient in a prone position, which significantly improved oxygenation and facilitated the resolution of secretions and inflammation. Additionally, magnetically navigated nasoenteric tube placement effectively mitigated high aspiration risks, ensuring smooth enteral nutrition and enhancing treatment safety and efficacy. This approach offers new therapeutic insights and clinical experience for managing similar complex cases in the future.

## 7. Conclusion

This case successfully addressed the challenges of contraindications to PPV and nutritional support, demonstrating therapeutic potential in complex clinical scenarios. The medical team prioritized patient safety, communicated effectively with the family, weighed the pros and cons, and made transparent and well-considered decisions. However, it should be emphasized that this method is based on a single case experience and is not generalizable. Larger studies are needed before it can become a standard recommendation. This case offers valuable practical experience for similar cases; however, further research is essential to validate its broader applicability.

## Acknowledgments

We express our gratitude to the illustrator Yang Yu for providing the magnetic navigation image in Figure 4A.

## Author contributions

**Conceptualization:** Qianrong Ding, Huan Liu.

**Data curation:** Qianrong Ding, Jing Yang, Lican Zhao, Huan Liu.

**Formal analysis:** Lican Zhao, Huan Liu.

**Funding acquisition:** Yongming Tian.

**Investigation:** Lican Zhao, Huan Liu.

**Methodology:** Qianrong Ding, Lican Zhao, Huan Liu.

**Project administration:** Qianrong Ding, Jing Yang, Huan Liu, Yongming Tian.

**Resources:** Jing Yang.

**Visualization:** You Yuan, Jing Yang.

**Writing – original draft:** Qianrong Ding, You Yuan.

**Writing – review & editing:** Yongming Tian.
